# Food Waste Materials as Low-Cost Adsorbents for the Removal of Volatile Organic Compounds from Wastewater

**DOI:** 10.3390/ma12244242

**Published:** 2019-12-17

**Authors:** Maria Agostina Frezzini, Lorenzo Massimi, Maria Luisa Astolfi, Silvia Canepari, Antonella Giuliano

**Affiliations:** Department of Chemistry, Sapienza University of Rome, Piazzale Aldo Moro, 5, 00185 Rome, Italy; mariaagostina.frezzini@uniroma1.it (M.A.F.); l.massimi@uniroma1.it (L.M.); marialuisa.astolfi@uniroma1.it (M.L.A.); silvia.canepari@uniroma1.it (S.C.)

**Keywords:** volatile organic compounds, adsorption, environmental remediation, food waste materials, wastewater treatment

## Abstract

The aim of this work was to study the potential of food waste materials (banana peel, potato peel, apple peel, lemon peel, coffee waste, decaf coffee waste, grape waste, and carob peel) as low-cost adsorbents for the removal of aliphatic and aromatic volatile organic compounds (VOCs) from wastewater. The ability of examined food waste materials to adsorb VOCs from synthetic multi-component standard solutions was evaluated and the examined food waste materials showed high removal efficiency. Performances of coffee waste, grape waste, and lemon peel were detailed by using Trichloroethylene and p-Xylene in mono-component standard solutions. The adsorption capacity of the three selected food wastes was determined by using linear Langmuir and Freundlich isotherm models. Two errors functions, average percentage error (APE) and the chi-square test (χ^2^), were used for isotherm optimization prediction. Freundlich isotherm well described the adsorption of VOCs on the considered materials. According to the obtained results, a multilayer, physical, and cooperative adsorption process was hypothesized, particularly evident when the VOCs’ concentrations are high. This was confirmed by the high adsorption efficiency percentages (*E%* > 80%) of VOCs from a real polluted matrix (urban solid waste leachate), containing high concentrations of total organic content.

## 1. Introduction

Volatile organic compounds (VOCs), aliphatic and aromatic, are a wide class of organic pollutants extensively released in the environment from a variety of anthropogenic sources such as fuel storage and transport, industrial operations [1], manufacture and storage of paints, and combustion processes [2]. Most of the VOCs can cause direct and indirect harmful effects to humans as well as the environment. Moreover, they represent one of the main causes of chronic contamination, especially in industrialized countries, since their chemical and physical properties enable them to persist in the environment. Chlorinated volatile organic compounds (Cl-VOCs) and aromatic hydrocarbons (BTEXs; benzene, toluene, and xylenes) are ubiquitous contaminants frequently detected in the environment. Therefore, human exposure to Cl-VOCs and BTEX is very common; they enter the body through multiple routes, such as inhalation, ingestion, and dermal absorption [3,4,5]. The toxicity of these compounds is increased by the fact that VOCs are strongly lipophilic with a capacity to concentrate in fat deposits by determining a long-term exposure [6,7]. Since the beginning of the 20th century, Cl-VOCs were extensively used as solvents for processes such as dry cleaning, production of pesticides, paints, and refrigerants [8,9]. Among them, Trichloroethylene is one of the most prevalent and persistent contaminants detected in polluted environments [10] and has been classified as group 2A by IARC (International Agency for Research on Cancer), as probably carcinogenic to humans [11]. In fact, exposure to Trichloroethylene seems to cause harmful effects on central nervous system and on the immune and endocrine systems in adults [12], and may also contribute to certain types of cancers in adults and children [9].

BTEXs, which have been found mainly in sites contaminated by petrol, gasoline, and petrochemical products [13,14], are well known carcinogens [12,15] that can adversely affect various human organs [16] by causing, for example, irritation of the eyes and reduction of bone marrow function [17]. Hence, it appears indispensable to eliminate these hazardous and mutagenic chemicals from the environment [18].

Several methods for wastewater decontamination from organic pollutants are reported [19,20,21,22]. Among these, the adsorption process has been efficaciously used for the removal of organic as well as inorganic compounds from wastewater because of its safety and ease in operation [23,24,25,26]. One of the most used materials to adsorb organic pollutants is activated carbon [25,27,28,29]; however, it has some disadvantages, such as the high cost and the environmental problems related to the regeneration and disposal of its waste [30,31]. During the past years, several studies have been focused on the evaluation of low-cost and environmentally friendly technologies for the removal of pollutants from wastewater. Most of these studies regarded low-cost materials, mainly biosorbents, which were found to be able to reduce availability and concentration of some organic compounds (mainly industrial organic dyes, polycyclic aromatic hydrocarbons, and phenolic compounds) [19,21,30,31,32,33,34].

Among the biosorbents, food waste materials appeared to be efficient for the removal of inorganic pollutants from wastewaters [35,36,37,38,39,40,41]. In a recent paper [42], food waste adsorbents were tested on a real polluted matrix and they showed high removal efficiency of various heavy metals. Biosorption by food waste materials has several benefits such as low production costs [43], free availability, and possible reuse of the biosorbents [44]. Moreover, it is essential to use waste as raw material for new products and new applications [45] as a renewable source of biomass [35]. The use of such residual materials might become a viable alternative for wastewater treatment with associated environmental and economic benefits [46].

Since the high danger of volatile organic compounds for humans and the environment along with the scarce amount of studies regarding the biosorption of VOCs by food waste materials, the evaluation of the performances of these materials as VOCs adsorbents constitutes an interesting issue that still should be investigated.

In this study, eight food waste materials (banana peel, potato peel, apple peel, lemon peel, coffee waste, decaf coffee waste, grape waste, and carob peel) were evaluated as potential low-cost biosorbents for the removal of VOCs from wastewater. With this aim, their adsorption efficiency and capacity of aliphatic/aromatic VOCs from multi-component and mono-component synthetic solutions and from a real polluted matrix were explored.

## 2. Materials and Methods

### 2.1. Preparation of the Biosorbents

**Figure A1:**
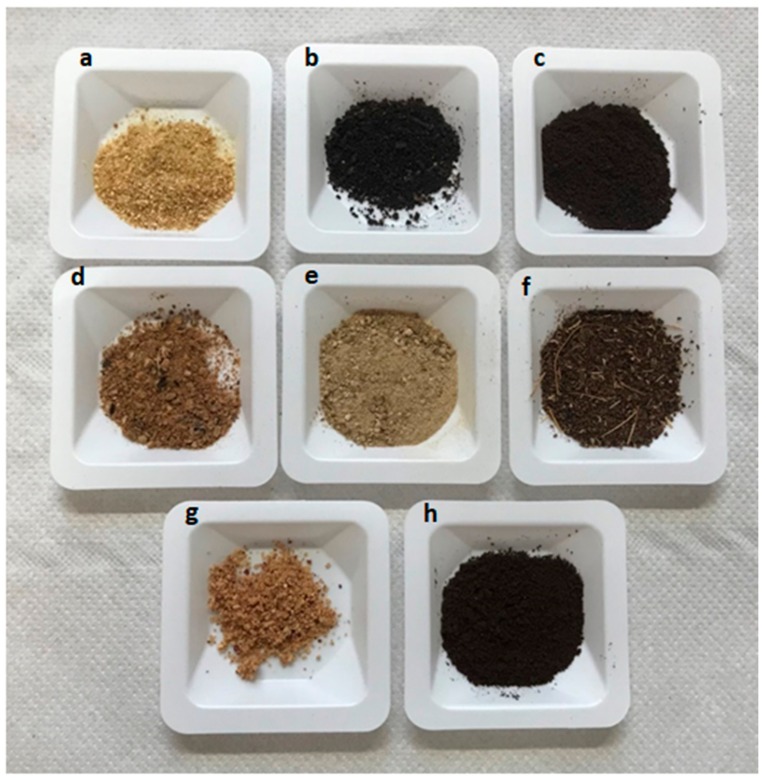
Food waste powders: (**a**) lemon peel, (**b**) grape waste, (**c**) coffee waste, (**d**) carob peel, (**e**) potato peel, (**f**) banana peel, (**g**) apple peel, (**h**) decaf coffee waste.

Food waste powders (n = 8) were employed as low-cost adsorbents: banana peel, potato peel, apple peel, lemon peel, coffee waste, decaf coffee waste, grape waste and carob peel (Figure A1, Appendix A). Preparation of the biosorbents followed the procedure detailed in a recent study [42]. Briefly, food waste materials were sun-dried for a week, grinded with a mortar and sieved to retain particles sized between 0.25 and 0.125 mm. The obtained powders were washed three times with deionized water (produced by an integrate water purification system; Arioso UP 900; Industrial Scientific Corporation, Pittsburg, PA, USA), dried at 55 °C for 48 h in a vacutherm oven (Heraeus VT 6025; Kendro Laboratory Products, Hanau, Germany), and then weighed on an analytical balance (Gibertini Europe 60; Gibertini Elettronica Srl, Milano, Italy).

### 2.2. Adsorption Experiments

First, removal efficiency of organic pollutants was explored by exposing each biosorbent to a synthetic multi-standard solution containing both aliphatic and aromatic VOCs (Trans-1,2-Dichloroethene, Dichloromethane, 1,1-Dichloroethene, 1,1-Dichloroethane, Chloroform, Carbon tetrachloride, Trichloroethylene, 1,2-Dichloropropane, 1,1,2-Trichloroethane, Tetrachloroethylene, Dibromochloromethane, 1,1,2-Tetrachloroethane, Bromoform, Methyl tert-butyl ether, Benzene, Toluene, Chlorobenzene, Ethylbenzene, m,p-Xylene, o-Xylene, 1,3-Dichlorobenzene, 1,4-Dichlorobenzene, 1,2-Dichlorobenzene). In these preliminary trials, 100 mg of each adsorbent was exposed to 10 mL multi-standard solutions at 20 µg/L by using 20 mL glass vials, hermetically sealed. Multi-standard solutions were obtained by diluting 2 mg/L multi-component solution, prepared by mixing proper aliquots of aliphatic and aromatic standard stock solutions (SPEX CertiPrep, Metuchen, New Jersey, USA) in methanol.

The adsorption kinetic was assessed by keeping both the mass of each waste material (100 mg) and the VOCs concentration (20 μg/L) constant, while increasing exposure time from 1 h to 24 h (1, 2, 3, 4, 5, 6, and 24 h).

All the samples were analysed to determine the equilibrium concentration (*C_e_*), which is the amount of adsorbate remaining in solution after adsorption processes. Multi-component standard solutions without biosorbent were always treated together with each sample set, to trace and control possible VOCs’ concentration variability due to loss by evaporation during treatment; VOCs’ concentration of these solutions was used as initial concentration (*C_0_*).

The performances of the food waste materials as biosorbents in a real polluted matrix were tested by exposing 100 mg of each food waste sorbent to 10 mL of an urban solid waste leachate. The same procedure described for the synthetic multi-standard solutions was applied to perform the exposure experiments and to determine *C_0_* and *C_e_*. The total organic content (TOC) of this solution was measured by a TOC analyzer (TOC-VCSH; Shimadzu Corporation, Kyoto, Japan).

The pH of both multi-standard solutions and real polluted matrix, with and without biosorbents, was measured using a pH meter (Criston MicropH 2002, Crisonb Instruments, Barcelona, Spain).

According to results obtained from the just described preliminary trials, two selected target VOCs were chosen for deepening the study of adsorption behavior in synthetic mono-component solutions. Trichloroethylene and p-Xylene were representatives of aliphatic and aromatic classes, respectively. In these trials, adsorption capacity was calculated for three selected food wastes by varying VOCs’ concentration in the range 25–2000 µg/L and by keeping constant at 100 mg the adsorbent amount. Coffee waste, grape waste, and lemon peel were chosen for this experimental procedure as biosorbents, with reference to a recent paper in which all the considered food waste materials were characterized in detail by scanning electronic microscopy (SEM), Fourier transform infrared spectroscopy (FTIR), and principal component analysis (PCA) [40]. Specific surface areas were estimated by analyzing 1 g of each adsorbent by krypton gas physisorption (Porosimeter 3Flex 3500; Micromeritrics, GA, USA). Obtained values were included in the range 0.1–0.5 m^2^/g. These values were close to the detection limit of the technique and the high uncertainties (about 0.05 m^2^/g) did not allow a reliable comparison among materials.

Aromatic and aliphatic VOCs considered in this work were identified by comparison with the analytes registered in NIST Mass Spectral Library (1.6 NIST MS Software Version 2.3) of static headspace gas chromatography coupled to mass spectrometry (SHS-GC-MS) (probability of more than 70%) and quantified.

### 2.3. GC Analysis

Static headspace gas chromatography coupled to mass spectrometry (SHS-GC-MS; QHSS-40 Headspace sampler, QUMA Elektronik & Analitik GmbH, Wuppertal, Germany; Varian 431-GC and Varian 210-MS, Varian, California, CA, USA) was used for all the VOCs’ analyses. After having chosen the exposure time, the glass vials containing the solutions (with or without the biosorbent) were maintained under stirring for 23 min at the constant temperature of 80 °C, then 1 µL of the vapor phase was injected in a capillary column (VF-624 30 m × 0.25 mm × 1 µm; stationary phase composition: equivalent of a 6% cyanopropyl-phenyl/94% dimethylpolysiloxane) with a Hamilton syringe (Hamilton Company, Bonaduz, Switzerland). Helium was used as carrier gas at the flow rate of 1 mL/min. The temperature was kept at 35 °C for 2 min, then it was increased to 50 °C at the rate of 6 °C per minute and, lastly, to 180 °C at the rate of 12 °C per minute. The total duration of the chromatographic run was 16 min.

Chromatograms were registered in selective ion monitoring mode (SIM) by selecting specific mass to charge ratios (m/z) for each analyte. The conditions employed to mass spectrometry and selected fragments and retention times of the considered VOCs and are summarized in Table A1 and Table A2 (Appendix A), respectively.

Final volume of each analysed sample was 10 mL and all the experiments were performed in triplicate. External standard solutions were used for quantification.

### 2.4. Calculation of Adsorption Efficiency and Adsorption Capacity

Adsorption efficiency (*E%*) was calculated by the following equation:

(1) $$E\%=\;\left[\frac{\left(C_{0}\;-\;C_{e}\right)}{C_{0}}\right]\cdot 100\%,$$

where *C_0_* (µg/L) is the inlet VOCs’ concentration and *C_e_* (µg/L) is the equilibrium concentration after the adsorption process.

Regarding real polluted matrix, for identified species, the adsorption efficiency was roughly estimated by comparing the peak areas in the presence (*A_e_*) and in the absence (*A_0_*) of the biosorbent ($E\%=\;\left[\frac{\left(A_{0}-\;A_{e}\right)}{A_{0}}\right]\cdot 100\%$) after adsorption processes.

Adsorption capacity *Q_e_* (µg/g) was determined by following the procedure described in Wang et al. [47], using a mass equilibrium equation:

(2) $$Q_{e}=\;\frac{\left[\left(C_{0}\;-\;C_{e}\right)\cdot V\right]}{m},$$

where *V* is the volume of solution (L) inside the vial and *m* is the amount (g) of food waste powder.

### 2.5. Adsorption Isotherms

The characterization of adsorption isotherms is considered one of the most appropriate methods for the assessment of the adsorbent capacity [47]. Adsorption isotherms play an important role in the predictive modelling procedures to understand what is going on during adsorption process and indicate how molecules, subjected to adsorption, distribute themselves between liquid and solid phases at equilibrium [48,49]. Linear regression analysis was used to obtain adjustable isotherm parameters. Table 1 summarizes the linear forms of Langmuir and Freundlich isotherm models used for this adsorption study. Although Langmuir isotherm model could be linearized into four different types, the reported Langmuir equation, defined Type I, is the most popular linear form used in available literature [50].

For Langmuir isotherms, *C_e_* (mg/L) is the equilibrium concentration after adsorption process, *Q_e_* (mg/g) is the adsorption capacity, *q_m_* (mg/g) and *K_L_* (in L/mg) are sorption equilibrium constants. The constant *K_L_* is related to the free energy of adsorption and indicates affinity between the VOCs and the adsorbents [51].

In Freundlich isotherm, *K_F_* (mg/L) and *n* (dimensionless) are both constants indicative of sorption capacity and sorption intensity, respectively [52,53]. In particular, *n* indicates the favorability of the adsorption process [52]; a close to unit *n* value represents good adsorption capacity, meaning VOCs’ adsorption is favorable [52]. The evaluation of the correlation coefficients (*R^2^*) is useful to predict which model best matches with the experimental data. In addition, the average percentage error (*APE*) and chi-square test (χ^2^) values were calculated in order to verify the validity of the isotherm models. APE and χ^2^, defined ad distribution functions, indicate the fit between the experimental and the predicted values of the adsorption capacity [54] and were used for isotherm optimization prediction [49]: if data from the model are similar to the obtained experimental data, the function values will be small number. APE and χ^2^ were calculated as follows:

(3) $$APE\;\left(\%\right)=\left[\frac{\sum_{i=1}^{N}\left|\frac{\left(Q_{e,exp}-Q_{e,pre}\right)}{Q_{e,exp}}\right|}{N}\right]\cdot 100,\;\chi^{2}=\;\overset{N}{\underset{i=1}{\sum }}\frac{{\left(Q_{e,exp}-Q_{e,pre}\right)}^{2}}{Q_{e,pre}},$$

where *Q_e,exp_*, *Q_e,pre_* are experimental and predicted adsorption capacity, respectively; *N* is the number of observations in the experimental data.

## 3. Results and Discussion

### 3.1. Adsorption Efficiency from Multi-Component Solutions

**Figure A2:**
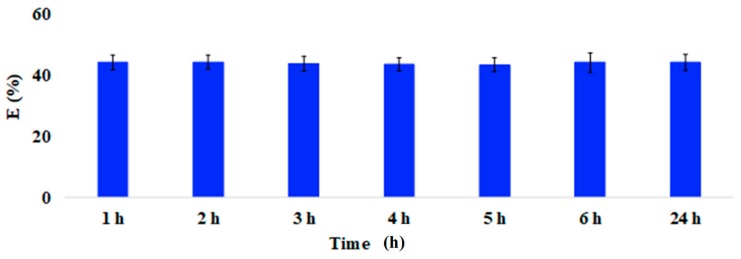
Adsorption efficiency (E%; 100 mg of coffee waste) of Trichloroethylene after 1, 2, 3, 4, 5, 6 and 24 h from preparation. Mean ± SD of three replicates is reported. The adsorption kinetic was assessed by keeping both the mass of each waste material (100 mg) and the VOCs’ concentration (20 µg/L) constant, while increasing exposure time from 1 h to 24 h (1, 2, 3, 4, 5, 6, and 24 h).

Preliminary tests on synthetic multi-component solutions were performed in order to verify the general behavior of the considered food waste materials as VOCs’ biosorbents. First, the rate of the adsorption process was evaluated by exposing the materials to a synthetic mixture of aromatic and aliphatic VOCs for increasing times (1, 2, 3, 4, 5, 6, and 24 h). As shown in Figure A2 (Appendix A), preliminary tests confirmed that 1 h contact time was enough to reach a steady-state VOC concentration, regarding both the liquid/solid and the liquid/gas interfaces. Consequently, all the successive samples were analysed after one hour from preparation.

Adsorption efficiency of each examined food waste material (100 mg) from a mixture of aliphatic and aromatic VOCs at 20 µg/L is reported in Table 2. Data show that all the considered biosorbents exhibited some adsorption properties towards organic species; the efficiency of adsorption was very low for some compounds and for some investigated adsorbents, with some exceptions. Coffee and decaf coffee waste appeared to be the most efficient for the removal of most of the organic compounds. For example, among the aromatic compounds, p-Xylene, o-Xylene, 1,2-Dichlorobenzene, 1,3-Dichlorobenzene, and 1,4-Dichlorobenzene were adsorbed in high percentages (*E%* > 50%); within the aliphatic compounds, Trichloroethylene, Tetrachloroethylene, and 1,1,2-Tetrachloroethane showed more than 40% of adsorption efficiency in presence of coffee and decaf coffee waste. Moreover, coffee waste was able to adsorb more than 90% of the Trans-1,2-Dichloroethene in multi-component solution. Apple peel seemed to be efficient for 1,1,2-Tetrachloroethane (*E%* > 50%) and for 1,2-Dichlorobenzene, 1,3-Dichlorobenzene and 1,4-Dichlorobenzene (*E%* > 30%). Lastly, it is worth noting that also grape waste adsorption efficiency is considerable for 1,2-Dichlorobenzene, 1,3-Dichlorobenzene and 1,4-Dichlorobenzene (*E%* > 40%) and 1,1,2-Tetrachloroethane (*E%* > 50%).

In general, looking at Table 2, aromatic compounds seem to be more retained than the aliphatic; moreover, coffee waste, decaf coffee waste, grape waste, and banana peel showed fairly good adsorption efficiency for both aliphatic and aromatic compounds.

Considering the efficiency of the examined low-cost adsorbents for the removal of VOCs from synthetic solutions, other experimental tests were conducted to confirm the potential of the investigated food waste materials to adsorb organic compounds from a real polluted matrix. The chosen real polluted matrix had a very high total organic content (TOC = 7500 mg/L), and a spontaneous pH of 6.5. In Table A3 (Appendix A), pH values both of multi-standard synthetic solutions and real polluted matrix are reported, with and without each adsorbent.

The real polluted matrix (a leachate of urban solid waste) contained a wide variety of VOCs; only some of them were identified by comparison with NIST library (>70% probability). The estimated adsorption efficiencies for these compounds are reported in Table 3. These data indicate that most of organic compounds is strongly retained by these adsorbents. In general, all the materials exhibited very good adsorption properties towards most of the VOCs and the results confirmed that coffee, decaf coffee, and grape waste were the most efficient biosorbents. Estimated efficiencies were much larger than those calculated in synthetic solutions. For example, coffee waste adsorption efficiency (*E%*) of Trichloroethylene was 43 ± 2 and 99 ± 12; lemon peel showed a Chlorobenzene *E%* of 10 ± 1 and 86 ± 12 and grape waste showed a m, p-Xylene *E%* of 34 ± 3 and 96 ± 5 in synthetic and real polluted matrix, respectively. This behavior may be due to the different VOCs’ concentration range in real polluted sample compared to the synthetic one, and seems to indicate a cooperative mechanism of adsorption, meaning an increase in the amount of pollutants adsorbed when initial VOCs’ concentration was increased. Coffee waste, decaf coffee waste, and grape waste were confirmed to be the most efficient biosorbents for the removal of both aliphatic and aromatic VOCs.

### 3.2. Adsorption Capacity from Synthetic Mono-Component Solutions

Adsorption processes were further investigated by studying the adsorption isotherms of selected biosorbents and VOCs. Two selected target VOCs were chosen for deepening the study of adsorption behavior in synthetic mono-component solutions. Trichloroethylene and p-Xylene were chosen as representative of aliphatic and aromatic classes, respectively. In these trials, VOCs’ concentration was varied in the range of 25–2000 µg/L, keeping constant at 100 mg the adsorbent amount. Adsorption capacity was calculated for three selected food waste biosorbents (coffee waste, grape waste, and lemon peel) according to the results obtained from a previous study, in which all the considered food waste materials were analysed by scanning electronic microscopy (SEM) and Fourier transform infrared spectroscopy (FTIR) [42]. In this study, FTIR spectra were elaborated by principal component analysis (PCA) to group the food waste materials in different clusters, according to functional groups present on the adsorbents’ surfaces. First cluster included orange and lemon peel, and was characterized by OH of alcohols, phenols and carboxylic acids; CH, COC, CN, PO of polysaccharides and C=C of lipids and lignin moieties; lemon peel was chosen as representative of this cluster.

Second cluster included coffee and decaf coffee waste, apple, and banana peel, and was characterized by the highest amount of CH bonds of methyl and methylene groups of lipids; coffee waste was chosen as representative of this cluster. Third cluster included grape waste and carob peel and was characterized by the lowest amount of the identified functional groups; grape waste was chosen as representative of this cluster.

**Figure 1:**
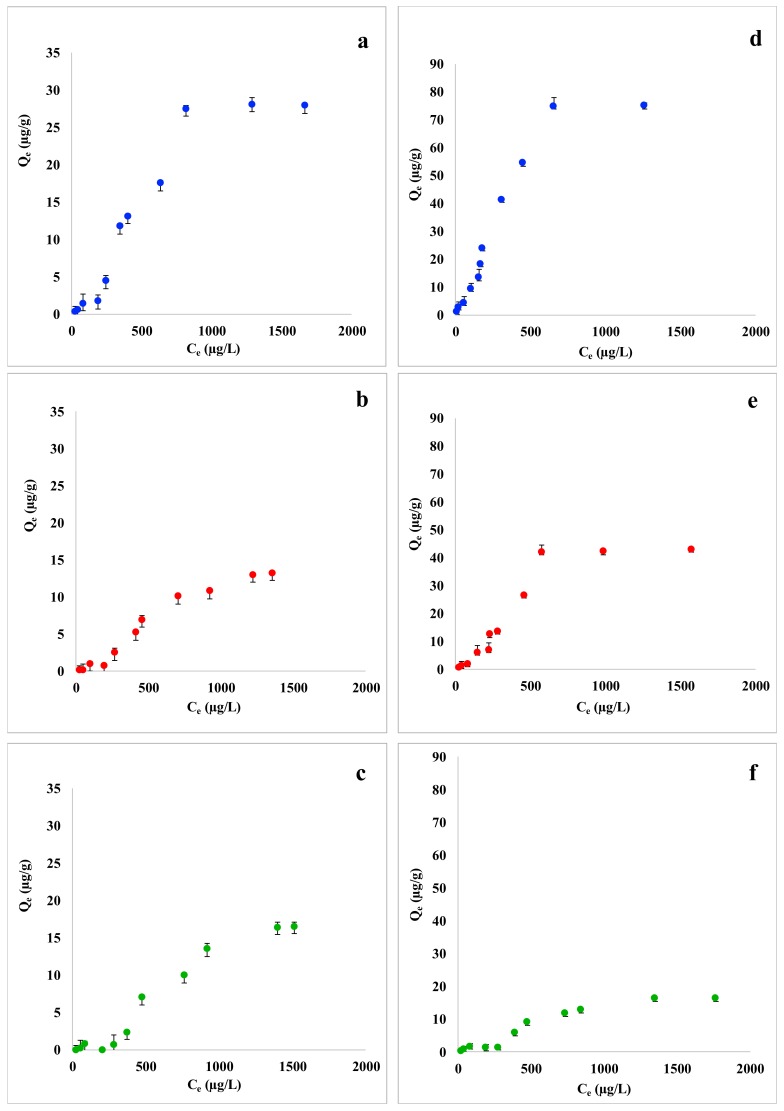
Comparison of adsorption capacity (Q_e_, µg/g) isotherms of (**a**) coffee waste, (**b**) grape waste, and (**c**) lemon peel with Trichloroethylene (from 25 to 2000 µg/L; 100 mg of each biosorbent) and (**d**) coffee waste, (**e**) grape waste, and (**f**) lemon peel with p-Xylene (from 25 to 2000 µg/L; 100 mg of each biosorbent). Mean ± SD of three replicates is reported.

Adsorption capacity of coffee waste, grape waste and lemon peel towards Trichloroethylene and p-Xylene is graphically represented in Figure 1. Plots a, b, and c show the adsorption curves of coffee waste, grape waste, and lemon peel for Trichloroethylene. As expected, the adsorption capacity of the three low-cost sorbents increases with increasing the pollutant exposure concentration. Trichloroethylene was adsorbed with the highest values of *Q_e_* (µg/g) in presence of coffee waste, while grape waste and lemon peel were found to be less efficient. Adsorption capacity curves show a quite complex trend and seem to indicate that the adsorbent saturation was reached at the tested conditions. Figure 1 (plots d, e, f), shows the adsorption curves of coffee waste, grape waste, and lemon peel for p-Xylene. The trend of adsorption curves of p-Xylene with all the three biosorbents was in general quite similar to that seen for Trichloroethylene: an increase of adsorption capacity is observed with the increase of pollutant’s concentration. p-Xylene adsorption capacity appeared to be higher than that of Trichloroethylene for all the examined food waste materials. For example, coffee waste adsorption capacity can be recalled: the highest Q_e_ value reached with Trichloroethylene and p-Xylene was 30 µg/g and 80 µg/g, respectively.

These results agree with those obtained from the adsorption experiments in multi-component standard solutions. Coffee waste was found to be the most efficient biosorbent for the removal of both Trichloroethylene and p-Xylene. The most plausible explanation comes from the chemical-physical properties of Trichloroethylene, p-Xylene, and coffee waste composition. FTIR spectra of coffee waste showed peaks of asymmetric and symmetric stretching of hydrophobic C-H bonds of methyl and methylene groups, attributed to the presence of lipids that are available in coffee samples in large amount [42,55]. Considering that Trichloroethylene and p-Xylene are both well-known lipophilic compound [56,57], a significant adsorption on lipid-rich coffee waste was expected. Moreover, according to the SEM micrographs, coffee waste has very porous surface [42] that allows it to adsorb a higher amount of pollutants from wastewater. Finally, lemon peel turned out to be the least efficient adsorbent for Trichloroethylene and p-Xylene with the highest *Q_e_* recorded value of 10 µg/g and 20 µg/g, respectively, probably because of its less porous surface and the presence of polar functional groups [42].

### 3.3. Adsorption Isotherms

**Figure 2:**
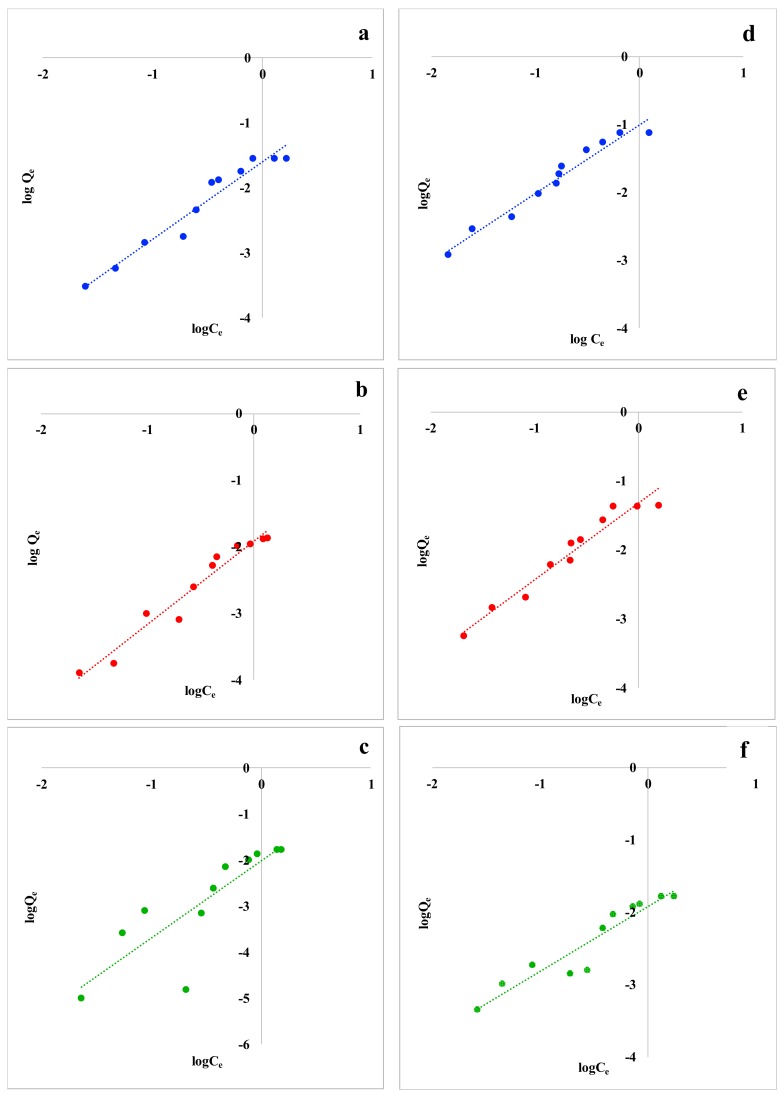
Freundlich isotherms for Trichloroethylene with (**a**) coffee waste, (**b**) grape waste, and (**c**) lemon peel; Freundlich isotherms for p-Xylene with (**d**) coffee waste, (**e**) grape waste, and (**f**) lemon peel.

Sorption equilibrium data for the two VOCs and the three food waste materials were fitted to the Langmuir and Freundlich isotherm models. Although many adsorption models were developed and applied in the literature [58,59], the Langmuir and Freundlich adsorption models are frequently used to describe adsorption mechanism. Although nonlinear isotherm forms are recommended in the field of batch adsorption research to avoid errors resulting from simple linear regression [49,60], in this study, linearized isotherm models were adopted due to the mathematical simplicity [51] and to immediately obtain semi-quantitative isotherm constants. Equilibrium data did not fit Langmuir model for both the studied VOCs and for the considered biosorbents. Since Langmuir isotherm cannot be applied in this work, the corresponding data are not reported. Linearized Freundlich adsorption isotherms for Trichloroethylene and p-Xylene are reported in Figure 2.

The constants obtained by applying the Freundlich isotherm model are summarized in Table 4. The high correlation coefficients (*R^2^* ≥ 0.9) obtained by plotting log*Q_e_* versus log*C_e_* for both Trichloroethylene and p-Xylene indicate that these adsorption processes are well represented by the Freundlich model. Lemon peel showed a lower correlation, but it’s still significant.

Moreover, the values of *n* and *K_F_* of the Freundlich model indicate good adsorption intensity and capacity, respectively: *n* was close to unity for all the examined food waste adsorbents and for both Trichloroethylene and p-Xylene, indicating that VOCs adsorption is favorable [52]. Furthermore, the low values of *APE* and *χ^2^* in association with higher *R^2^* generate a satisfactory fit of Freundlich model to the experimental data, even if they are little higher for lemon peel. To explain these contrasting results, the adsorption processes simulated by the two models are to be considered. Langmuir model represents only monolayer adsorption processes [46] on the outer surface of the adsorbent, without considering any further adsorption [61]. Instead, Freundlich isotherm represents heterogeneous surfaces and sorbent systems [47,62] in aqueous systems [30], in which multilayer adsorption processes are included. According to the increase of the adsorption capacity with increasing of the VOCs’ concentration, a multilayer adsorption process can be hypothesized. This is also confirmed by the *n* values reported in Table 4. In fact, when *n* < 1, the presence of an isotherm called “solvent-affinity isotherm” is suggested [62]; this kind of isotherm represents an increase of the adsorption energy with increasing concentration of pollutants on the adsorbents’ surface. Thus, it can be assumed that aliphatic and aromatic VOCs are initially weakly detained by food waste materials through an adsorption on the active sites of the sorbents. At high concentrations, the active sites are completely occupied by the compounds and the adsorption of other pollutants is supported by Van der Waals forces with the molecules already adsorbed, making a physical adsorption within adsorbent layers. The result is an effective adsorption process within the adsorbent layers, caused by strong intermolecular attractions.

## 4. Conclusions

Food waste materials, that are available in large amount and at very low costs, appeared to be promising biosorbents for both aliphatic and aromatic VOCs in aqueous solutions. In general, the aromatic VOCs were better adsorbed than the aliphatic by most of the considered materials. This is confirmed by the high percentages of adsorption efficiency (*E%*).

Adsorption capacities (*Q_e_*) of three chosen food waste materials (coffee waste, grape waste, and lemon peel) with Trichloroethylene (chosen as representative of aromatic VOCs) and p-Xylene (chosen as representative of aliphatic Cl-VOCs) were investigated; coffee waste resulted the most efficient sorbent for the removal of both considered VOCs. This is probably due to the presence of lipids in coffee samples on which Trichloroethylene and p-Xylene compounds are efficiently adsorbed, due to their lipophilic nature.

Adsorption equilibrium isotherms showed that coffee waste is the most efficient material for both p-Xylene and Trichloroethylene adsorption. In all the examined systems, the sorption processes followed much more closely the Freundlich than the Langmuir isotherm model. This indicates a physical adsorption mechanism thanks to London-Van der Waals forces between solute and sorbent, through a cooperative adsorption mechanism, in which the already adsorbed molecules on the sorbent’s surface facilitate the adsorption of other molecules. This kind of physical adsorption may be particularly beneficial in field applications, because it gives the adsorbents an opportunity to have great adsorption performance at high pollutant concentrations.

Evaluation of food waste performances as biosorbents in field still needs further deepening, such as the effects of pH on removal capacity, the assessment of the effects of sorbents’ particle size, the investigation of repetitive adsorption desorption cycles for material reusability investigation, or even the comparison between other food waste materials’ adsorption capacity. Taking into account all the considerations, the results of this preliminary study suggest further investigations about the possible applicative use of these low-cost adsorbents for VOCs’ removal from wastewater.

In this context, the efficient adsorption capacity of food waste materials at high VOCs’ concentration was preliminarily confirmed by the excellent adsorption efficiencies obtained in a real polluted matrix.

## Figures and Tables

**Table 1:** Linear forms of Langmuir (Type I) and Freundlich isotherm models.

Isotherm Models	Linear
Langmuir	$\;\frac{C_{e}}{Q_{e}}\;=\;\frac{1}{\left(K_{L}\;\cdot \;q_{m}\right)}\;+\;\frac{C_{e}}{q_{m}}\;$
Freundlich	lnQe= lnKF + 1n · ln Ce

**Table 2:** Adsorption efficiency (*E%*; 100 mg of each biosorbent) of aliphatic and aromatic VOCs from multi-component synthetic solution (20 µg/L). Mean ± SD of three replicates is reported.

Adsorption Efficiency (*E%*) ± SD								
Aliphatic VOCs	Banana Peel	Potato Peel	Apple Peel	Lemon Peel	Coffee Waste	Decaf C. Waste	Grape Waste	Carob Peel
Trans-1,2-Dichloroethene	2.7 ± 0.4	0.6 ± 0.2	0.7 ± 0.4	1.6 ± 0.8	93 ± 18	7.6 ± 1.4	4.7 ± 1.6	2.3 ± 1.6
Dichloromethane	3.9 ± 0.2	5.7 ± 4.3	8.2 ± 3.2	7.1 ± 1.8	9.8 ± 0.7	13 ± 1	6.2 ± 1.9	3.7 ± 1.1
1,1-Dichloroethene	1.8 ± 0.3	1.1 ± 0.7	3.3 ± 0.8	3.1 ± 1.8	3.9 ± 0.5	12 ± 5	6.7 ± 1.2	1.7 ± 1.3
1,1-Dichloroethane	1.3 ± 0.8	5.6 ± 1.9	2.6 ± 0.2	13 ± 6	12 ± 2	16 ± 1	6.7 ± 1.7	3.1 ± 1.3
Chloroform	5.9 ± 5.4	7.2 ± 2.4	1.5 ± 0.5	5.1 ± 2.6	6.2 ± 2.3	13 ± 1	6.3 ± 1.1	7.7 ± 3.1
Carbon tetrachloride	39 ± 2	4.3 ± 1.9	11 ± 2	6.3 ± 0.3	17 ± 2	16 ± 5	37 ± 12	4.3 ± 1.7
Trichloroethylene	35 ± 1	16 ± 13	36 ± 1	44 ± 1	43 ± 2	51 ± 1	34 ± 2	36 ± 1
1,2-Dichloropropane	1.1 ± 0.2	0.9 ± 0.5	8.3 ± 6.4	4.7 ± 2.1	1.1 ± 0.2	16 ± 1	5.5 ± 3.2	3.8 ± 3.4
1,1,2-Trichloroethane	7.5 ± 6.5	5.4 ± 4.3	13 ± 5	14 ± 4	18 ± 4	25 ± 9	11 ± 1	2.5 ± 0.9
Tetrachloroethylene	17 ± 12	12 ± 4	11 ± 4	1.8 ± 0.9	35 ± 9	47 ± 10	42 ± 7	11 ± 2
Dibromochloromethane	43 ± 5	1.1 ± 0.8	2.5 ± 1.8	1.4 ± 0.8	13 ± 1	27 ± 3	11 ± 2	0.8 ± 0.3
1,1,2-Tetrachloroethane	39 ± 1	23 ± 2	57 ± 7	38 ± 1	73 ± 13	77 ± 1	51 ± 1	8.5 ± 1.8
Bromoform	57 ± 2	1.6 ± 0.9	17 ± 4	1.3 ± 0.6	19 ± 2	25 ± 2	25 ± 2	2.3 ± 0.7
Aromatic VOCs								
Methyl tert-butyl ether	1.8 ± 0.2	9.5 ± 1.9	1.8 ± 0.8	3.1 ± 2.3	10 ± 5	1.5 ± 1.4	1.5 ± 1.1	1.5 ± 1.1
Benzene	1.8 ± 0.8	2.8 ± 0.3	1.8 ± 0.2	1.3 ± 1.2	17 ± 1	19 ± 1	7.1 ± 0.5	5.9 ± 0.3
Toluene	12 ± 1	1.5 ± 1.1	8.5 ± 0.5	3.9 ± 0.3	31 ± 2	33 ± 2	19 ± 2	1.3 ± 0.8
Chlorobenzene	17 ± 1	5.5 ± 0.5	15 ± 1	10 ± 1	37 ± 1	42 ± 1	30 ± 1	2.5 ± 2.1
Ethylbenzene	7.1 ± 1.4	4.8 ± 1.8	9.5 ± 5.5	1.5 ± 0.7	28 ± 1	37 ± 3	24 ± 17	1.6 ± 0.4
m, p-Xylene	35 ± 1	12 ± 1	21 ± 1	15 ± 1	54 ± 1	56 ± 1	34 ± 3	3.3 ± 1.8
o-Xylene	28 ± 1	14 ± 3	24 ± 1	18 ± 4	57 ± 1	58 ± 1	33 ± 5	13 ± 2
1,3-Dichlorobenzene	35 ± 2	12 ± 1	34 ± 1	19 ± 2	67 ± 1	73 ± 2	48 ± 4	12 ± 1
1,4-Dichlorobenzene	43 ± 2	14 ± 1	36 ± 2	20 ± 1	68 ± 1	71 ± 1	47 ± 1	24 ± 1
1,2-Dichlorobenzene	36 ± 1	10 ± 2	37 ± 1	20 ± 2	67 ± 1	73 ± 2	47 ± 2	13 ± 2

**Table 3:** Adsorption efficiency (E%; 100 mg of each biosorbent) of VOCs from real polluted matrix of urban solid waste leachate. Mean ± SD of three replicates is reported.

Adsorption Efficiency (*E%*) ± SD								
Aliphatic VOCs	Banana Peel	Potato Peel	Apple Peel	Lemon Peel	Coffee Waste	Decaf C. Waste	Grape Waste	Carob Peel
1,1-Dichloroethene	20 ± 5	38 ± 9	60 ± 10	79 ± 18	91 ± 16	89 ± 5	87 ± 6	71 ± 11
Dichloromethane	85 ± 11	85 ± 15	90 ± 18	96 ± 15	97 ± 9	97 ± 5	97 ± 10	93 ± 7
Chloroform	24 ± 9	13 ± 6	54 ± 12	83 ± 14	92 ± 10	93 ± 5	90 ± 9	69 ± 16
Trichloroethylene	44 ± 8	39 ± 6	56 ± 13	98 ± 16	99 ± 12	96 ± 6	97 ± 10	86 ± 9
Dibromochloromethane	8.6 ± 2.3	3.2 ± 1.4	47 ± 14	84 ± 10	95 ± 11	93 ± 2	89 ± 8	67 ± 6
Aromatic VOCs								
Toluene	31 ± 9	14 ± 7	59 ± 10	79 ± 18	93 ± 9	91 ± 9	89 ± 15	62 ± 8
Chlorobenzene	45 ± 8	15 ± 6	67 ± 18	86 ± 12	97 ± 9	93 ± 5	93 ± 7	71 ± 7
m, p-Xylene	64 ± 9	27 ± 11	78 ± 10	91 ± 14	99 ± 10	98 ± 2	96 ± 5	77 ± 10
o-Xylene	72 ± 11	26 ± 13	78 ± 12	92 ± 17	93 ± 9	95 ± 8	97 ± 9	78 ± 9
1,3-Dichlorobenzene	77 ± 13	37 ± 15	93 ± 18	98 ± 10	98 ± 7	98 ± 2	97 ± 11	87 ± 7

**Table 4:** Parameters of Freundlich isotherm for Trichloroethylene and p-Xylene.

	*K_F_* (mg/L)	*n*	*R^2^*	APE (%)	χ^2^
**Trichloroethylene**					
Coffee waste	0.026	0.83	0.99	2.9	3.1 × 10^-5^
Grape waste	0.012	0.79	0.99	5	1.8 × 10^-5^
Lemon peel	0.009	0.61	0.97	22	1.3 × 10^-5^
**p-Xylene**					
Coffee waste	0.11	0.98	0.99	0.91	1.2 × 10^-5^
Grape waste	0.051	0.89	0.98	0.86	3.4 × 10^-6^
Lemon peel	0.012	1.1	0.97	8	1.1 × 10^-3^

## Appendix A

**Table A1:** Conditions employed to mass spectrometry (Varian 210-MS ion trap mass spectrometer with electron ionization).

Acquisition Method	Ion Trap Temperature (°C)	Transfer Line Temperature (°C)	Vacuum Manifold Temperature (°C)
SIM (Single Ion Monitoring)	150 °C	180 °C	80 °C

**Table A2:** GC-MS parameters for VOCs analyses.

VOCs	Retention Time (min)	Fragments (m/z)	Acquisition Range (m/z)
**Aliphatic VOCs**			
**Trans-1,2-Dichloroethene**	4.50–4.90	61–96–98	55–100
**Dichloromethane**	3.90–4.50	49–84–86	49–90
**1,1-Dichloroethene**	3.20–3.90	61–96–98	60–100
**1,1-Dichloroethane**	4.90–5.50	63–65–27	60–90
**Chloroform**	5.50–6.55	83–85–47	45–90
**Carbon tetrachloride**	6.70–6.90	117–121	75–125
**Trichloroethylene**	7.40–7.95	130–95–97	55–135
**1,2-Dichloropropane**	7.95–8.25	63–62–27	60–80
**1,1,2-Trichloroethane**	9.70–9.86	97–83–61	44–170
**Tetrachloroethylene**	9.86–10.10	166–164–129	160–175
**Dibromochloromethane**	10.10–10.75	129–127	80–132
**1,1,2-Tetrachloroethane**	11.78–11.87	81–82.8–83.8	80–85
**Bromoform**	11.73–12.02	173–171	75–180
**Aromatic VOCs**			
**Methyl tert-butyl ether**	1.11–2.10	44.8–45.8	40–48
**Benzene**	6.9–7.08	78–77	45–80
**Toluene**	9.15–9.45	91–92	60–100
**Chlorobenzene**	10.75–11.00	112–77–114	48–120
**Ethylbenzene**	11.00–11.13	91–106	85–110
**m-Xylene**	11.30–11.73	91–106	70–120
**p-Xylene**	11.30–11.73	91–106	70–120
**o-Xylene**	11.13–11.30	91–106	70–120
**1,3-Dichlorobenzene**	12.75–13.71	146–148–111	70–150
**1,4-Dichlorobenzene**	13.71–13.85	146–148–111	70–150
**1,2-Dichlorobenzene**	13.85–15.00	146–148–111	70–150

**Table A3:** pH of real polluted matrix and synthetic multi-component standard solution with and without the eight food waste materials.

Food Waste Materials	Real Polluted Matrix	Synthetic Multi-Component Standard Solution
Without adsorbents	6.5	7
Banana peel	5.5	5.8
Potato peel	6.4	7.5
Apple peel	5.9	4.7
Lemon peel	4.7	4.7
Coffee waste	6.2	5.9
Grape waste	6.1	7.1
Carob peel	5.9	5.3
Decaf Coffee waste	6.5	6.2
